# Enhancing neurosurgical navigation operation flow management through personal digital assistant technology: a prospective cohort study

**DOI:** 10.3389/fpubh.2024.1408378

**Published:** 2024-05-31

**Authors:** YaQin Quan, ManHong Zhang, HongMing Ji, Rui Cheng

**Affiliations:** ^1^Department of Anesthesiology Surgery, Shanxi Provincial People’s Hospital, Taiyuan, Shanxi, China; ^2^Department of Neurosurgery, Shanxi Provincial People’s Hospital, Taiyuan, Shanxi, China

**Keywords:** personal digital assistant, neurosurgery, patient satisfaction, nursing care, digital health

## Abstract

**Purpose:**

This prospective cohort study aims to evaluate the impact of digital health technology especially Personal Digital Assistants (PDA) in neurosurgical procedure management, focusing on surgical safety check accuracy, efficiency, and patient satisfaction.

**Methods:**

The study included 211 neurosurgical cases from January to December 2022. The control group of 106 patients followed traditional verification methods, while the experimental group of 105 patients used PDA. The PDA system facilitated real-time data collection, verification, and transmission. The study compared both groups in terms of check times, accuracy rates, and patient satisfaction, and used multivariate regression to assess the impact of baseline parameters on these outcomes.

**Results:**

The study found that the experimental group using the PDA system reduced the average verification time by approximately 8 min, achieving 100.0% accuracy in preoperative and postoperative checks, significantly better than the control group (91.5% pre- and post-operation). Multivariate regression confirmed a 48.1% reduction in postoperative verification time due to the PDA system (*p* < 0.001), with the model showing high explanatory power (R^2^ = 0.911). Other examined factors, including patient age and nurse experience, had no significant effects. Similarly, the PDA’s introduction markedly improved verification accuracy, with no significant impact from other variables (*p* = 0.010).

**Conclusion:**

The application of the PDA system in neurosurgical operations significantly enhanced the accuracy and efficiency of surgical safety checks, reduced nursing errors, optimized nursing workflows, and improved patient satisfaction. These results provide valuable insights for the application of PDA technology in high-risk medical fields, demonstrating potential of digital health tools in enhancing surgical safety and efficiency.

## 1 Introduction

The advancement of digital health technologies, such as Personal Digital Assistants (PDAs), offers transformative potential for public health (1), particularly in high-stress medical environments like neurosurgical operating rooms. In neurosurgical operations, ensuring patient safety is the primary task (2). The “Guide To Operating Room Nursing Practice” (3) emphasize several aspects of surgical patient safety, such as accurate patient identification and enhanced safety verification processes. However, relying on traditional surgical process management methods faces numerous challenges in the busy and stressful operating room environment.

Manual verification processes depend on the operations of medical staff, which are not only time-consuming and inefficient, but also prone to committing errors in tense or high-pressure situations (4). Additionally, the slow update of information further increases the risk of errors. While electronic health record (EHR) systems offer a platform for storing and verifying patient information, the unique setting of an operating room presents challenges in accessing these details in real time and significant challenges persist in achieving full interoperability across acute care settings (5), especially without mobile devices. Furthermore, delayed updates in electronic records may not reflect the most recent changes in patient conditions. Periods of EHR downtime are operationally disruptive and pose significant risks to patients (6). Finally, barcode scanning technology, which relies on specific scanning equipment and labels, has limitations in information capacity and is prone to device malfunctions or misuse (7, 8). In recent years, some innovative attempts have been made to address these challenges. For example, the World Health Organization (WHO) “Safe Surgery Saves Lives” initiative and the surgical safety checklist have been adopted by many hospitals, such as the University of California, Los Angeles (UCLA) Health System, which has been using an extended surgical safety checklist since 2008 (9). These checklists ensure the correctness and safety of surgery through the “pause” segment before skin incision. Although these advancements have achieved certain effects in improving the safety and effectiveness of surgical process management, unique challenges still exist in neurosurgical operations.

Given these general concerns in surgical safety, it becomes evident that innovative solutions are needed. This study introduces the use of PDA in conjunction with existing hospital information systems, exploring their application in neurosurgical operation process management. PDAs, through the hospital’s wireless network, enable real-time data collection, verification, and transmission, offering advantages such as improved accuracy and efficiency of information verification (10), as well as strong mobility and ease of use. The application of this technology has the potential to overcome the limitations of traditional methods. It can enhance operating room work efficiency and further promote the digital transformation of hospitals (11).

The integration of PDA systems in medical nursing has demonstrated substantial effectiveness across various departments, with their benefits in enhancing nursing quality, efficiency, and patient satisfaction being corroborated by multiple studies. In emergency department, it was found that using PDAs significantly reduced the time for data gathering (6 min and 13 s per patient with PDA vs. 9 min and 12 s with paper) and decreased the rate of errors (12). Furthermore, a study evaluated the impact of using a PDA with a diary function on adherence to interferon β-1b treatment in Multiple Sclerosis (MS) patients, comparing it to a traditional paper diary. Results indicated that male patients showed a 10.0% lower drop-out rate when using a PDA, and the use of a PDA with a reminder function improved adherence to the injection schedule by an average of 24.50 injections over 24 months, suggesting that electronic diaries with reminders can enhance adherence to MS therapy (13). Regarding the reduction of medication errors and the enhancement of nursing care efficiency, the study revealed that nursing students utilizing PDAs performed medication-related tasks with greater accuracy and speed compared to those relying on textbooks. This underscores the effectiveness of PDA technology in nursing education, highlighting its potential as a valuable tool for training future healthcare professionals (14). In orthopaedic surgical practice, PDAs have been recognized for their proficiency in data acquisition, analysis, and scheduling, credited to their bright screens, user-friendly interfaces, and compact designs. Their integration with forms management tools like Pendragon Forms and compatibility with diverse computer database programs has improved data management in clinical environments. This facilitates record-keeping and research, while simultaneously reducing secretarial workload (15). However, there is a lack of studies specifically focusing on the application of PDA systems in neurosurgery and analyzing their mechanisms of action.

This study focuses on how PDAs can improve verification work and process management by neurosurgical operating room medical staff. It aims to provide new ideas and methods for hospital departments in policy-making and to demonstrate how PDA technology can enhance the safety and efficiency of the operating room, while exploring the underlying mechanisms of its function. Our research results are beneficial for improving neurosurgical operation processes and increasing patient safety.

## 2 Materials and methods

### 2.1 Study subjects

This preoperative cohort study was approved by the Institutional Review Board (Approval no. 202279). We initially assessed 902 patients who underwent navigational surgeries in our neurosurgery department from January to December 2022. Inclusion criteria included patients with non-frontal temporal parietal tumors and those with similar surgical histories further exclusions were made for patients with significant cardiac or renal diseases, other severe comorbidities, or known allergies to surgical drugs or materials used in the study. Patients who are unwilling to share their medical records were also excluded. This filtering process resulted in a cohort of 211 patients.

**Figure 1:**
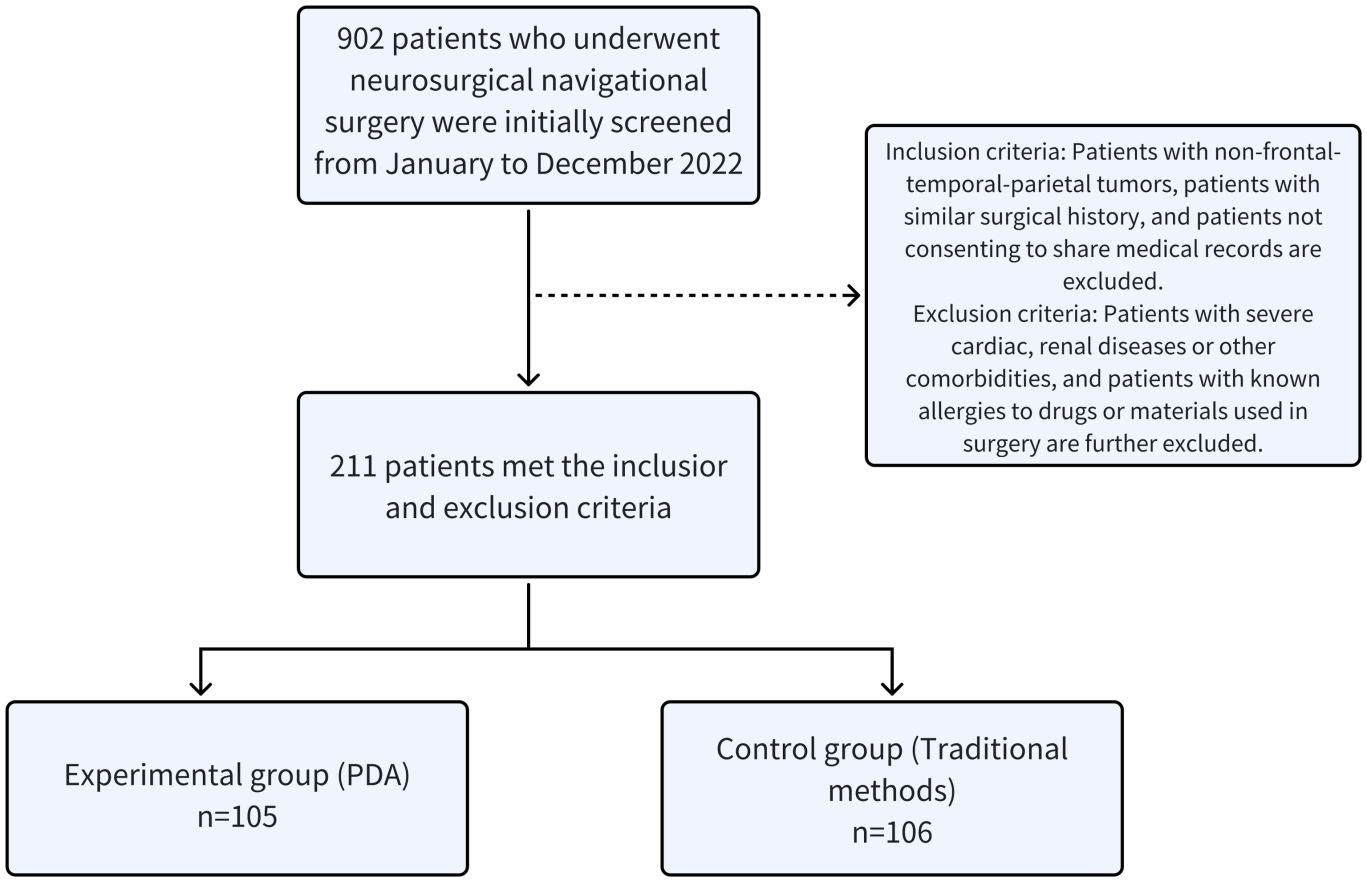
Inclusion and exclusion flow chart of study subjects.

In this study, we implemented simple randomization to allocate participants into two groups, ensuring an unbiased selection process. This randomization was conducted using a computer-generated random number sequence, which provided each participant with an equal probability of assignment to either the experimental or control group. The experimental group consisted of 105 patients who were managed perioperatively with (PDAs, while the control group included 106 patients who received traditional manual verification methods). This method of random allocation aimed to ensure equitable distribution of baseline characteristics such as age, gender, and severity of illness across both groups, as depicted in Figure 1. Patients in the experimental group ranged in age from 13 to 84 years, with a mean age of 50.72 ± 14.71 years; the control group comprised patients aged 15 to 82 years, with a mean age of 54.32 ± 13.33 years. We performed t-tests and Chi-squared tests to assess comparability between the groups regarding gender ratios, severity of illness, and other critical characteristics, confirming no significant differences (all *p*-values >0.05). Additionally, the operating room nurses participating in the study were comparable in terms of educational background, job position, gender, and other fundamental demographics, further ensuring that our findings could be attributed to the intervention rather than confounding variables.

### 2.2 Implementation process

In this study, the control group employed traditional manual verification methods, which involved multiple stages including patient entry into the surgery room, pre-anesthesia, pre-operation, post-operation, patient admission to the recovery room, and patient discharge from the recovery room. At each stage, the circulating nurse, anesthesiologist, and surgeon collaboratively verified patient information to ensure the accuracy of the patient’s identity and surgical details. The specific implementation process was as follows: Prior to entering the room, the circulating nurse would first inquire about the patient’s name and check the patient’s wristband hospital ID to confirm their identity; before anesthesia, the patient’s name, department, age, method of anesthesia, surgery date, and surgical site were verified against the surgical safety management list; before surgery, the circulating nurse and the surgeon would double-check the patient and surgical method, surgical site, with checks on related surgical information both before (prior to the use of navigation) and after the surgery; upon the patient’s admission to and discharge from the recovery room, the circulating nurse would once again verify the patient’s name, wristband information, surgical method, surgeon, and method of anesthesia. To measure the time spent on safety verification for neurosurgical patients, the control group was assisted by nurse aides in timing, ranging from the start of verification upon entering the surgery room to the patient’s discharge from the anesthesia recovery room/out of the room.

The experimental group utilized mobile PDAs to log into the surgical patrol management system, which consisted of nine modules: preoperative visit, disinfection goods application, entering the surgery room, pre-anesthesia check, silent check, surgery completion, leaving the surgery room, entering the recovery room, and leaving the recovery room/patient discharge. All these modules involved automated information collection and verification.

The specific implementation process of logging into the surgical patrol management system with mobile PDAs was:

(1) Preoperative visit: operating room nurses, equipped with mobile PDAs, would visit patients in the ward on the day before surgery. By scanning patients’ QR-coded badges, they logged into the surgical patrol management system and activated the preoperative visit module. Upon scanning the patients’ QR-coded wristbands, the system’s patient identity recognition module would automatically document the time and personnel of the visit, concurrently displaying the patients’ relevant surgical information. Following patient identity confirmation and surgical details review, nurses would instruct patients or their relatives to access preoperative health education through a WeChat QR code specifically designed for the department. Emphasizing explanations tailored to the surgical approach, the system then auto-generated a visitation record for each patient. After the visitation, the system calculated visitation and return visit rates, drawing on the volume of visits and surgeries. Nurses could review these records and perform self-audits using desktop computers. Subsequently, the head nurse would relay any particular observations from these visits, ensuring awareness of any specific patient requirements for the subsequent day’s surgeries, thereby ensuring a state of readiness and initiating a comfortable and secure preoperative phase for the patients.
(2) Disinfection supplies application module: when entering the disinfection supplies application module, circulating nurses scan the QR codes on sterile instrument packs with the PDA, capturing essential data. The system then automatically links each patients’ names and bed locations to the scanned packs, cataloging details such as item names, personnel responsible for sterilization, times of sterilization, and expiration dates. Packs confirmed as sterile and within their use-by date are flagged as successfully utilized, facilitating subsequent reviews of procedural results and supporting the traceability of quality controls.
(3) Operating room module: in this module, the PDA is utilized to scan QR-coded wristbands of surgical patients, badges of circulating nurses, and QR codes specific to the operating rooms. This scanning process automatically generates critical surgical data including the patient’s name, age, gender, department, bed number, hospital ID, infectious disease status, designated surgery, surgical site, type of surgery, surgeon, planned surgery time, circulating nurse’s name, room number, and entry time. This meticulous procedure ensures that patients slated for elective surgeries are accurately guided to their assigned operating rooms, effectively eliminating the risk of patient or surgical misplacements.
(4) Pre-anesthesia verification module: this module involves using the PDA to scan the surgical patients’ QR-coded wristbands and the QR-coded badges of circulating nurses, anesthesiologists, and surgeons. This procedure automatically compiles essential surgical details such as the patient’s name, age, gender, department, bed number, hospital ID, infectious disease information, planned surgery name, surgical site, type of surgery, proposed anesthesia method, and the names of the surgeon and anesthesiologist. It also records the scheduled time for surgery and the initiation time for anesthesia. This rigorous verification step is integral to ensuring the accuracy of critical information for elective surgery patients prior to the commencement of anesthesia.
(5) Silent verification module: within this module, the PDA is employed to scan QR-coded wristbands of surgical patients as well as the QR-coded badges of circulating nurses, anesthesiologists, and surgeons. In addition, it captures an audio log of relevant patient information. This action leads to the automatic generation of a detailed patient profile, encompassing the patient’s name, age, gender, department, bed number, hospital ID, infectious disease details, the intended surgical procedure, method, surgical site, type of surgery, planned anesthesia technique, and the names of both the surgeon and anesthesiologist, along with the scheduled surgery start time. The module incorporates a ‘time-out’ process to verify the accuracy of this information, thus ensuring the reliability of crucial details for patients scheduled for elective surgeries, while also preventing any errors related to the surgical procedure or site.
(6) Surgery completion verification module: in this module, scanning of the surgical patients’ QR-coded wristbands and the QR-coded badges of circulating nurses, anesthesiologists, and surgeons is conducted using the PDA. This scan automatically collates extensive patient data, encompassing the patient’s name, age, gender, department, bed number, hospital ID, infectious disease specifics, surgery name (with a manual entry and modification feature), anesthesia method, and the names of the lead surgeon, anesthesiologist, and surgical nurse, culminating in the recorded time of surgery completion. This data is then promptly displayed on the electronic screens in the waiting room, effectively updating the family members of surgical patients about the ongoing status and orderly progression of the surgery.
(7) Exiting the operating room verification module: this module activates upon the PDA’s scanning of the surgical patients’ QR-coded wristbands and the QR-coded badges of the circulating nurses, anesthesiologists, and surgeons. It meticulously compiles a complete patient profile, which includes details such as the patient’s name, age, gender, department, bed number, hospital ID, infectious disease information, both the intended and actual surgeries conducted (with options for manual adjustment), anesthesia method, surgical site, type of surgery, names of the lead surgeon and anesthesiologist, surgical nurse, and the start and end times of the surgery, including the anesthesia completion time, surgery room’s name, and the moment of exiting the operating room. The system rapidly calculates and updates this time-sensitive information, which is then relayed to the electronic screens in the waiting room, keeping the patient’s family informed of the nearing end of the surgical procedure.
(8) Recovery room entry verification module: in this module, surgical patients’ QR-coded wristbands and the QR-coded badges of circulating nurses, anesthesiologists, and surgeons are scanned using the PDA. This procedure efficiently aggregates extensive patient details, including their name, age, gender, department, bed number, hospital ID, infectious disease profile, planned and actual surgeries performed (with an option for manual adjustments), anesthesia method, surgical site, surgery type, and the names of the primary surgeon, anesthesiologist, circulating nurse, and recovery room nurse. Additionally, it records the completion times of the surgery and anesthesia, the name of the surgery room, and the moment the patient enters the recovery room. As patients enter the recovery room, this array of information is automatically relayed to the waiting room’s electronic screens, offering updates to the families about the patient’s postoperative state, including their consciousness and limb functionality.
(9) Recovery room exit/patient discharge verification module: this module is initiated by scanning the surgical patients’ QR-coded wristbands and the QR-coded badges of circulating or recovery room nurses, anesthesiologists, and surgeons. It seamlessly compiles an exhaustive patient profile, covering their name, age, gender, department, bed number, hospital ID, infectious disease information, conducted surgeries, anesthesia method, surgical site, type of surgery, and the names of the attending surgeon, anesthesiologist, circulating nurse, and recovery room nurse. This profile also includes the start and end times of the surgery and anesthesia, and the timings of the patient’s exit from the recovery room or discharge. This data is promptly displayed on the electronic screens in the waiting room, keeping the patient’s family abreast of their overall postoperative condition, such as their awareness and physical state. Marking the last step of verification in the operating room, this module ensures a comprehensive closed-loop management of the entire surgical process, updating and documenting the progress of the surgery in real-time, thus safeguarding patient safety and enhancing the quality of surgical care.

### 2.3 Follow-up observation variables and evaluation of PDA system effectiveness

In the follow-up phase, the study focused on several key observation variables: the time and accuracy of surgical safety checks, shifts in satisfaction levels, incidence of adverse events, adherence to and accuracy of surgical safety check protocols, and the compliance and accuracy rates in using sterile items. These variables covered the preoperative, intraoperative, and postoperative phases, with the goal of extensively contrasting the differences between conventional methods and the PDA system application. The study specifically examined aspects such as the reduction in time taken for verification, enhancements in verification accuracy, improvements in both medical staff and patient satisfaction, a decrease in nursing errors and disputes, a reduction in the number of complaints, and the accurate utilization of sterile items in surgical procedures. The PDA verification group employed mobile computing devices to automatically record the time of surgical safety checks, mirroring the control group’s approach. This timing commenced with the verification start upon entering the surgical room and concluded with the patient’s exit from the anesthesia recovery room. These collected data points are instrumental in comprehensively evaluating the PDA system’s efficacy in improving the efficiency and safety of neurosurgical procedure management.

### 2.4 Statistical analysis

This study utilized SPSS 26.0 software for data processing and analysis. Prior to conducting the main analyses, a preliminary sample size calculation was performed to ensure sufficient power to detect meaningful differences and effects within our data. Based on previous study (12) and a power analysis (using PASS 15.0), we aimed to achieve a power of 80% (*β* = 0.2) and a significance level of α = 0.05 and assumed an expected effect size of 1 min for key comparisons in verification time. To ensure the robustness of our study design, a sensitivity analysis was also conducted, adjusting for variations in effect size (±0.5 min from the initial estimate).For quantitative data, such as verification times and satisfaction scores, a normality test was first conducted to ascertain the distribution characteristics of the data. If the data were normally distributed, they were presented as mean ± standard deviation (*M* ± SD), and t-tests were used to evaluate differences between groups. For categorical data, such as adverse event rates and protocol adherence rates, the data were expressed as *n* (%) and analyzed using the test.


$$\chi^{2}$$

Although the baseline characteristics of the study subjects were balanced between the two groups, potential interactions between different factors could still influence key study outcomes (16, 17). Therefore, to further explore the cumulative impact of various factors on critical research endpoints like verification times and accuracy, this study employed multiple linear regression analysis. This analysis considered predictive variables such as the gender and age of patients and nurses, nurses’ educational level and years of experience, and the application of the PDA, to assess their impact on surgical verification times and accuracy. The construction of the model was based on the statistical significance of each predictive variable’s coefficient and evaluated the overall fit of the model, such as the R^2^. In addition, model diagnostics were conducted to check for compliance with linear regression assumptions, including the independence and homogeneity of residuals.

Furthermore, for significant results, the effect size (Cohen’s d ) was reported to understand the practical significance of the findings more comprehensively. All statistical tests were set at a significance level of *p* < 0.05. The results of the study were visualized through appropriate graphs (such as bar charts and box plots) to intuitively reveal data distributions and comparisons between groups.

## 3 Results

### 3.1 Baseline characteristics

This study enrolled 211 participants, divided into a control group of 106 individuals and an experimental group of 105. A notable finding was the significant reduction in average verification time by approximately 8 min within the experimental group that utilized the PDA system. This effect size markedly exceeds the largest effect size of 1.5 min that had been used for the sensitivity analysis. The actual sample sizes for both the experimental and control groups substantially surpassed the estimated requirement to detect a 1-min effect size with 80% power at a 0.05 alpha level, which was 64 participants per group. This robust sample size endows the study with more than sufficient power, thereby minimizing the risk of a Type II error and reinforcing the statistical significance of the reduction in verification time.

Demographic characteristics including the age of patients, years of experience of the nurses, and their educational levels were analyzed and compared between the two groups. The results, as shown in Table 1, indicated no significant differences between the groups for age of patients (*p* = 0.061), years of experience of nurses (*p* = 0.416), and educational levels (*p* = 1.000), with similar frequency distributions observed. For the gender ratio of nurses, where minor discrepancies were noted [men: 2 (1.9%) in the control group and 4 (3.8%) in the experimental group], the Fisher Exact Test was applied due to the low frequency of male nurses, yielding a *p*-value of 0.445. This methodological adjustment ensures robustness in our statistical analysis, addressing the limitations associated with small sample sizes in certain categories. The educational level of nurses showed high uniformity, with the majority holding a bachelor’s degree.

**Table 1:** Patient demographics and characteristics (*N* = 201).

Variable	Control group	Experimental group	*Z or* $\chi^{2}$ *(P)*
	*n* (%) or *M* ± SD	*n* (%) or *M* ± SD	
Patient age	54.32 ± 13.33	50.72 ± 14.71	−1.876 (0.061)^a^
Nurse working years	12.60 ± 2.23	12.93 ± 2.76	−0.814 (0.416)^a^
Gender of patient			0.571 (0.450)^b^
Men	49 (46.2%)	54 (51.4%)	
Women	57 (53.8%)	51 (48.6%)	
Gender of nurse			(0.445)^c^
Men	2 (1.9%)	4 (3.8%)	
Women	104 (98.1%)	101 (96.2%)	
Educational level of nurse			(1.000)^c^
Undergraduate	104 (98.1%)	103 (98.1%)	
Post-graduate	2 (1.9%)	2 (1.9%)	

a Denotes the application of the Mann–Whitney U test.

b Signifies the use of the Chi-square test.

c Represents the Fisher’s Exact Test.

### 3.2 Impact of the PDA system on safety check time

**Figure 2:**
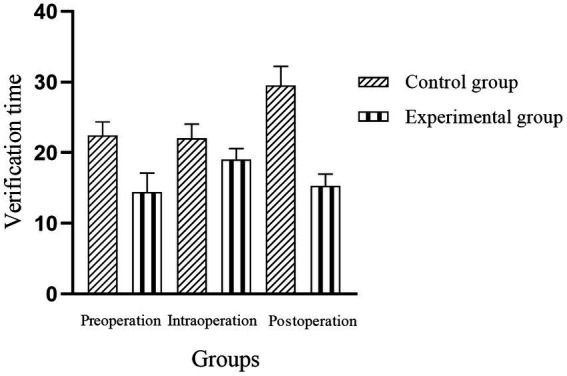
Comparison of traditional verification vs. PDA system in neurosurgical patient safety check duration (unit: minutes, data presented as *M* ± SD).

In the assessment of surgical safety check times, different statistical approaches were applied based on the distribution characteristics of the data. For intraoperative and postoperative times, confirmed as normally distributed through the Shapiro–Wilk test, t-tests demonstrated significant efficiency improvements. Specifically, intraoperative times showed a decrease from 22.03 ± 2.03 min in the control group to 19.01 ± 1.56 min in the experimental group (t = 12.11, *p* < 0.05), with a Cohen’s d value of 1.61, indicating a large effect size. Similarly, postoperative times decreased from 29.52 ± 2.69 to 15.33 ± 1.66 min (t = 46.06, *p* < 0.05), with an even larger effect size (Cohen’s d = 2.46). For preoperative times, which were non-normally distributed, a non-parametric test was applied. A significant reduction in time was observed for 22.41 ± 1.93 to 14.41 ± 2.72 min (Z = −12.37, p < 0.05). These results indicate that the use of the PDA system, in comparison to traditional methods, had a significant and larger effect size during these stages (Figure 2).

### 3.3 Impact of the PDA system on the accuracy of verification

In the experimental group using the PDA system, the rates of standardized execution and accuracy of checks in critical stages such as pre-anesthesia, pre-operation, and post-operation were close to or reached 100.0%, significantly higher than those in the control group. Specifically, in the pre-anesthesia phase, the accuracy rate of the experimental group was 99.1%, noticeably higher than the 95.3% of the control group. However, this difference was not statistically significant (*p* = 0.212), suggesting that despite numerical differences, due to sample size or other factors, it did not reach statistical significance. In the pre-operation and post-operation phases, the experimental group achieved 100.0% accuracy, compared to 91.5% in the control group. This difference was statistically significant (both *p* = 0.003), indicating that the use of the PDA system significantly improved the accuracy of verifications during these stages.

### 3.4 Impact of PDA system on satisfaction, nursing errors, verification accuracy and sterile item usage

#### 3.4.1 Satisfaction

In the experimental group, patient satisfaction reached 100.0%, showing an increase compared to 97.2% in the control group. Although this increase was statistically significant (χ^2^ = 15.18, *p* = 0.001), its clinical significance might be relatively limited since the satisfaction level in the control group was already quite high.

#### 3.4.2 Nursing errors, disputes, or complaints

No nursing errors, disputes, or complaints were reported in the experimental group during the study period, while the control group reported a 0.9% incidence of nursing errors (as shown in Table 2). This suggests that the PDA system may help reduce nursing errors, but given the low occurrence rate of errors, this conclusion requires more data for support. Future studies should increase the sample size for more precise statistical analysis.

**Table 2:** Accuracy of verification, satisfaction, nursing comments and sterile items usage.

Variable	Control group	Experimental group	$\chi^{2}$ *(P)*
	*n* (%) or *M* ± SD	*n* (%) or *M* ± SD	
Accuracy of verification			
Pre-operation	101 (95.3%)	104 (99.6%)	(0.212)^b^
Intra-operation	97 (91.5%)	105 (100.0%)	(0.003)^b^
Post-operation	97 (91.5%)	105 (100.0%)	(0.003)^b^
Satisfaction			15.175(0.001)^a^
Highly Satisfied	27(25.5%)	42(40.0%)	
Satisfied	43 (40.6%)	50 (47.6%)	
Somewhat Satisfied	33 (31.1%)	13 (12.4%)	
Dissatisfied	3 (2.8%)	0 (0.0%)	
Total satisfaction percent	97.2%	100.0%	
Nursing errors, disputes, or complaints			(1.000)^b^
Errors	1 (0.9%)	0 (0.0%)	
Disputes	0 (0.0%)	0 (0.0%)	
Complaints	0 (0.0%)	0 (0.0%)	
Sterile items usage			
Qualification	104 (98.1%)	105 (100.0%)	(0.498)^b^
Accuracy	103 (97.2%)	104 (99.1%)	(0.621)^b^

a Signifies the use of the Chi-square test.

b Represents the Fisher’s Exact Test.

#### 3.4.3 Sterile item compliance and correct usage rates

In the experimental group using the PDA system, the compliance rate for sterile items reached 100.0%, and the correct usage rate for surgical items was 99.1%. In contrast, the control group using traditional methods had a 98.1% compliance rate for sterile items and a 97.2% correct usage rate for surgical items. Although the experimental group scored higher on both metrics, statistical analysis indicated that these differences were not significant (*p* = 0.498 for sterile item compliance rate, *p* = 0.621 for correct usage rate of surgical items). This suggests that the differences between the observation and control groups may not be solely attributed to the use of the PDA system. Future research should explore other potential influencing factors.

### 3.5 Confounder-adjusted estimation of factors influencing verification time and accuracy

#### 3.5.1 Verification time

In the postoperative verification time analysis, a significant reduction of 48.1% was observed with the use of the PDA system, strongly suggesting a notable efficiency improvement in this phase with the PDA system. To further investigate the factors contributing to this reduction, especially considering potential influences such as patient age, gender, and the years of experience of nurses, a multiple regression analysis was conducted.

The multiple-variable regression analysis results revealed *F* = 348.076 with a corresponding *p* = 0.000, indicating the model’s overall statistical significance. The R^2^ value of the model was 0.911, with an adjusted R^2^ = 0.908, suggesting that the considered independent variables, including patient age, gender, nurse gender, and the application of the PDA, significantly impacted the postoperative verification time. Further analysis inferred that the substantial reduction in postoperative verification time was primarily attributable to the effective implementation of the PDA system (*p* = 0.000, see Table 3), while other factors did not significantly influence the reduction in verification time.

**Table 3:** Multiple regression analysis results: factors influencing postoperative verification time.

Variables	Unstandardized coefficient		Standardized coefficient	*t*	*p*	VIF
	B	Standard deviation	Beta			
Whether to apply PDA	−14.18	0.32	−0.95	−44.96	0.000	1.03
Patient age	−0.00	0.01	−0.00	−0.12	0.903	1.06
Patient gender	−0.37	0.31	−0.03	−1.17	0.243	1.02
Nurse gender	0.68	0.99	0.02	0.69	0.492	1.13
Nurse working years	−0.07	0.08	−0.02	−0.92	0.360	1.58
Nurse education level	0.56	1.37	0.01	0.41	0.681	1.44

#### 3.5.2 Verification accuracy

The introduction of the PDA system revealed a significant improvement in the accuracy of checks during key stages (pre-anesthesia, pre-operation, post-operation) in the experimental group, nearing or achieving 100.0%, which was markedly superior to the control group. To ensure that this improvement was not influenced by factors such as patient age, gender, or nurse work experience, a multiple regression analysis was conducted.

The multiple-variable regression analysis results indicated that the model was statistically significant overall (*F* = 2.889, *p* = 0.010). The *R*^2^ = 0.078, with an adjusted *R*^2^ = 0.051, suggesting that the considered predictive variables had a significant impact on the accuracy of verification. As shown in Table 4, *p*>0.05 for variables except the application of the PDA. This indicates that these factors did not significantly influence the improvement in verification accuracy, and the significant increase in accuracy can be primarily attributed to the effective use of the PDA system.

**Table 4:** Multiple regression analysis results: factors influencing postoperative verification accuracy.

Variants	Unstandardized coefficient		Standardized coefficient	*t*	*p*	VIF
	B	Standard deviation	Beta			
Whether to apply PDA	0.10	0.03	0.24	3.489	0.00	1.03
Patient age	0.00	0.00	0.13	1.892	0.06	1.06
Patient gender	0.01	0.03	0.03	0.373	0.71	1.02
Nurse gender	0.02	0.09	0.01	0.198	0.84	1.13
Nurse working years	−0.01	0.01	−0.14	−1.647	0.10	1.58
Nurse education level	0.19	0.12	0.12	1.540	0.13	1.44

## 4 Discussion

Considering the numerous challenges encountered in neurosurgical procedures during the perioperative period, such as critical patient conditions and consciousness impairments, this study developed and implemented a PDA system for use in neurosurgery. Compared to traditional methods, the PDA system significantly enhanced the accuracy and efficiency of surgical safety verification. For instance, the results indicate that the experimental group using the PDA system experienced an average reduction of about 8 min in verification time, with preoperative and postoperative verification accuracies reaching 100.0%, a statistically significant improvement (*p* = 0.05) compared to the control group (preoperative 91.5% and postoperative 91.5%).

To ensure the rigor of the study, strict inclusion, exclusion, and randomization processes were followed during participant enrollment, ensuring baseline balance. However, potential interactions between different factors might still affect the study outcomes (18). Therefore, this study used multiple regression analysis to explore whether factors such as patient age, gender, nurse gender, work experience, and educational level affected the reduction in postoperative verification time and improvement in verification accuracy. These factors were found to have almost no significant statistical impact, but the use of the PDA system was statistically significant in both aspects, indicating that its effective application can significantly reduce postoperative verification time and improve verification accuracy.

The PDA system automatically matches patient information by scanning QR-coded wristbands, eliminating traditional manual operations and record-keeping. In various record-keeping and nursing verification tasks, it enhances accuracy, reduces record time, and minimizes the possibility of human error. Its real-time updating feature ensures the timeliness of critical information during the surgical process, providing robust support for every key point of the surgery process — from patient identity verification to confirming the surgery site and method. This not only bolsters patients’ sense of security but also enhances patients’ satisfaction.

In traditional paper-based systems, preoperative visits often suffer from simplicity in content and non-standardization in record-keeping, such as the uniformity of oral explanations and inconsistencies in handwritten records (19). These limitations could lead to superficial patient understanding of the visit contents, non-standard writing, and arbitrary alterations. The PDA technology reduces errors and delays caused by handwritten records (20), significantly enhancing nursing work efficiency and accuracy. This improvement not only lightens the nurses’ workload (21), but also provides more personalized care for patients, enhancing their trust in the nursing staff (22). Additionally, the application of PDAs significantly reduced adverse events. The control group reported a 0.9% rate of nursing errors, whereas the experimental group successfully reduced it to 0.0%, highlighting the practical effectiveness of the PDA system in enhancing patient safety. The application of PDAs in preoperative visits showed marked advantages, improving the efficiency and quality of visits and increasing patient participation and satisfaction. This improvement is crucial for enhancing the quality and patient experience in the overall surgical preparation process.

In relation to technological advancements in healthcare, our findings align with those observed in other medical fields, which similarly emphasize the benefits of digital aids in clinical settings (12–14). For instance, a study by Siebert et al. (23) demonstrated how a mobile device application designed for pediatric cardiopulmonary resuscitation significantly reduced medication errors and the time to drug delivery. This application decreased medication errors by 68% and reduced drug preparation and delivery times by 45 and 40%, respectively, showcasing the potential of technology to enhance clinical efficiency and safety. Similarly, another research (24) highlighted the efficacy of the PedAMINES app, which reduced the time to drug preparation by 180 s and time to drug delivery by 177.3 s, while also eliminating 70% of medication errors, further corroborating the transformative impact of digital tools. In these departments, the application of PDAs has effectively alleviated the workload of medical staff while improving the quality and impact of patient care. Against the backdrop of the high risks and complexities of neurosurgical procedures, the application of the PDA system has demonstrated its unique value. Our study extends these findings to the neurosurgical context, where often involves delicate and high-risk procedures, demanding more stringent requirements for precise and timely information verification. In this study, the application of the PDA system in neurosurgery not only improved the accuracy and efficiency of surgical safety checks but also achieved 100.0% success in preoperative and postoperative verification accuracy. This result contrasts with the application outcomes in other departments like emergency and cardiology, highlighting its uniqueness and importance in the high-risk environment of neurosurgery.

Despite the advantages of the PDA system, its implementation faces challenges, including technological stability and user adaptability. Medical staff need appropriate training to proficiently use this new technology (25), and regular maintenance and updates are required to ensure the system’s efficient operation. A systematic review (26) underscores the need for this training by revealing significant gaps in digital health competencies among healthcare professionals, particularly in the psychological and emotional aspects of digital technology use. These competencies are crucial for not only using but also maximizing the potential of digital technologies in clinical settings. Future research should focus on the long-term impacts of using PDAs, particularly in enhancing patient safety and satisfaction. Additionally, considering the successful application of PDA technology in neurosurgery, its potential application in other medical fields is also worth exploring.

In assessing the generalizability of our study results, it is essential to consider the unique environment of neurosurgery and the specific challenges it presents, which PDA system in this study directly addresses. While our findings demonstrate significant improvements in surgical safety verification efficiency and accuracy within this high-risk context, the representativeness of our samples and the adaptability of the PDA system to other medical settings warrant further discussion. Our study’s rigorous randomization and adequate sample size strengthen the argument for its external validity within similar high-complexity medical fields. However, the technological and training requirements identified highlight potential barriers to broader implementation. The successful application of the PDA system in neurosurgery suggests its potential value in enhancing patient safety and care quality across various medical disciplines. Yet, the need for system customization to meet diverse departmental needs and continuous support for technological updates and staff training emphasizes the importance of future research. This research should explore the PDA system’s scalability, adaptability, and long-term impact on healthcare outcomes in different settings, aiming to provide a more nuanced understanding of its widespread applicability and the conditions under which it can best contribute to improving patient care and safety.

## 5 Conclusion

In summary, the application of PDA system in neurosurgery significantly improved the accuracy and efficiency of verifications, reduced nursing errors and verification time, optimized nursing workflows, and increased patient satisfaction. By enhancing safety and efficiency in complex surgical procedures, the PDA system contributes to reducing the overall healthcare burden, notably in terms of postoperative complications and hospital readmissions, which are critical factors in public health management. Furthermore, the success of the PDA system in neurosurgery underscores the potential of digital technology to revolutionize patient care in various medical settings, thereby supporting the broader public health objective of improving health care quality and accessibility. These study results not only provide a solid theoretical foundation for the further application of PDA technology in neurosurgery but also offer valuable insights for integrating similar digital health tools in other high-risk medical fields.

Future developments:

The proposed enhancement of IoT (Internet of Things) intelligent coverage aims to interconnect operating rooms, intensive care units, wards, nursing systems, and various devices across hospital areas. This will lead to a more integrated and comprehensive approach to data management.
Substantial improvements have been made to existing modules. For instance, the preoperative visit module now automatically recognizes data and generates patient-specific preoperative QR codes. Accessible via PDA, these QR codes contain essential information, ensuring that each surgical patient’s preoperative needs are precisely addressed. Moreover, the addition of a dynamic intraoperative feature to the surgical module, which links to patient monitors, provides real-time updates on vital signs – a critical factor in ensuring patient safety during surgery.
The development and integration of new modules further exemplify our commitment to enhancing patient care. On the day of surgery, patients are now equipped with medical-grade wireless devices that interface with the PDA to monitor key physiological parameters. This system enables anesthesiologists to make timely interventions, significantly boosting patient safety. Post-surgery, the implementation of a new module facilitates a smoother transition of patients back to the ICU or ward. This module, by sending surgery completion notifications to relevant units, prepares staff for postoperative care. Additionally, the introduction of a postoperative follow-up module, sending automated notifications on specific post-surgery days and at discharge, ensures a consistent follow-up rate and greatly improves patient satisfaction.

## Data availability statement

The raw data supporting the conclusions of this article will be made available by the authors, without undue reservation.

## Ethics statement

The studies involving humans were approved by Institutional Review Board of Shanxi Provincial People’s hospital. The studies were conducted in accordance with the local legislation and institutional requirements. Written informed consent for participation in this study was provided by the participants’ legal guardians/next of kin.

## Author contributions

YQ: Formal analysis, Methodology, Writing – original draft, Writing – review & editing. MZ: Writing – review & editing. HJ: Project administration, Writing – review & editing. RC: Project administration, Writing – review & editing.
